# The TRiC/CCT Chaperone Is Implicated in Alzheimer's Disease Based on Patient GWAS and an RNAi Screen in Aβ-Expressing *Caenorhabditis elegans*

**DOI:** 10.1371/journal.pone.0102985

**Published:** 2014-07-31

**Authors:** Eleonora Khabirova, Aileen Moloney, Stefan J. Marciniak, Julie Williams, David A. Lomas, Stephen G. Oliver, Giorgio Favrin, David B. Sattelle, Damian C. Crowther

**Affiliations:** 1 Department of Genetics, University of Cambridge, Cambridge, United Kingdom; 2 Department of Biochemistry, University of Cambridge, Cambridge, United Kingdom; 3 Cambridge Institute for Medical Research, University of Cambridge, Cambridge, United Kingdom; 4 MRC Functional Genomics Unit, Department of Physiology, Anatomy and Genetics, University of Oxford, Oxford, United Kingdom; 5 Sir William Dunn School of Pathology, University of Oxford, Oxford, United Kingdom; 6 Cambridge Systems Biology Centre, University of Cambridge, Cambridge, United Kingdom; 7 Department of Medicine, University of Cambridge, Cambridge, United Kingdom; 8 Department of Psychiatry and Neurology, MRC Centre for Neuropsychiatric Genetics, Cardiff University School of Medicine, Cardiff, United Kingdom; 9 The Wolfson Institute for Biomedical Research, University College London, London, United Kingdom; 10 Department of Medicine, University College London, London, United Kingdom; National Center for Geriatrics and Gerontology, Japan

## Abstract

The human Aβ peptide causes progressive paralysis when expressed in the muscles of the nematode worm, *C. elegans*. We have exploited this model of Aβ toxicity by carrying out an RNAi screen to identify genes whose reduced expression modifies the severity of this locomotor phenotype. Our initial finding was that none of the human orthologues of these worm genes is identical with the genome-wide significant GWAS genes reported to date (the “white zone”); moreover there was no identity between worm screen hits and the longer list of GWAS genes which included those with borderline levels of significance (the “grey zone”). This indicates that Aβ toxicity should not be considered as equivalent to sporadic AD. To increase the sensitivity of our analysis, we then considered the physical interactors (+1 interactome) of the products of the genes in both the worm and the white+grey zone lists. When we consider these worm and GWAS gene lists we find that 4 of the 60 worm genes have a +1 interactome overlap that is larger than expected by chance. Two of these genes form a chaperonin complex, the third is closely associated with this complex and the fourth gene codes for actin, the major substrate of the same chaperonin.

## Background

Once age is taken into account, the most powerful determinants of risk for Alzheimer’s disease (AD) are genetic; indeed, the heritability of the disorder is estimated to be 58–79% [2], [3]. In the clinic however, this strong genetic influence is not readily apparent because of the heterogeneous, multigenic inheritance of so-called sporadic AD. Such complexity has encouraged geneticists to focus on a small minority of AD cases that exhibit dominant Mendelian inheritance [4], [5]. Remarkably, these studies have shown that the bulk of the genetic lesions resulting in familial AD (FAD) directly influence the proteolytic processing of a single transmembrane protein called amyloid precursor protein (*APP*) [6]. The cleavage of *APP* by β-secretase and then **γ**-secretase results in the generation of a spectrum of peptides, known as amyloid β (Aβ), that are the major component of senile plaques in the brains of patients with AD. The **γ**-secretase is of particular interest because its cleavage of APP determines which amino acids constitute the C-terminus of Aβ. Normally, Aβ peptides are predominantly 40 amino acids in length (Aβ_40_), while 5–10% have a further 2 amino acids at the C-terminus (Aβ_42_). The bulk of cases of FAD can be accounted for by mutations that either increase the total amount of Aβ [7], [8], or favour the production of longer, more aggregation-prone, isoforms of the peptide [9]. FAD has been reported to be a consequence of variation in the sequence or copy number of either the *APP* gene, or the genes *PSEN1* or *PSEN2* encoding catalytic subunits of **γ**-secretase [7], [8]. Likewise, protection from AD has also been linked to a polymorphism in APP that interferes with β-secretase cleavage [10]. These data, supported by studies on sporadic AD patients [11], have given rise to the amyloid cascade hypothesis that places the aggregation of Aβ as the first step on the road to AD [6].

The advent of genome-wide association studies (GWAS) has made the investigation of multigenic disorders, such as sporadic AD, experimentally tractable. Using p<10^−7^ as an empirical cut off for genome-wide significance (the GWAS “white zone”), the first generation of multicentre AD GWAS [12], [13] reported four genes (*apoE*, *clusterin*, *PICALM*, and *CR1*) associated with disease risk. With the publication of the second generation of GWAS findings, the total number of risk genes is now twenty one [1], [14]. Notable absentees from these lists are any of the genes implicated in FAD. These findings may lead us to question the relevance of Aβ in the pathogenesis of sporadic AD and might indicate that the sporadic and familial forms are different diseases.

One explanation for the absence of genes such as *APP*, *PSEN1*, or *PSEN2* from the list of candidates identified by GWAS is that the studies were lacking sufficient power to detect small or rare contributions to risk [15]. Increased power could be achieved by recruiting more patients and controls and by increasing the density of SNP genotyping [15]. However, we have taken a parallel approach by including in our analysis not only the genes with genome-wide significance (GWAS “white zone”), but also GWAS results of borderline significance (GWAS “grey zone”) with p-values between <10^−5^. This larger list of genes will include false positives, that can safely be ignored, but equally there will be genes that carry risk and are involved in the pathogenesis of sporadic AD. Our approach has been to probe this larger group of GWAS white+grey zone genes using the results of a genome-scale screen in a model system that reports exclusively on the toxicity of the Aβ peptide. We postulate that if Aβ toxicity were distinct from sporadic AD then we would expect to see an overlap between the worm and human genes lists that is consistent with chance. However were the GWAS data reporting a role for Aβ in clinical AD we would expect to see a significantly increased overlap in the network of genes identified in the human and worm screens.

## Results

### Feeding RNAi to *C. elegans* modifies the Aβ-induced paralysis phenotype

We identified 7970 human protein-encoding genes that have orthologues in *C.elegans* and we used a subset of the Ahringer *C. elegans* RNAi library [16] to systematically knock down the transcript levels of each of these genes. The phenotypic consequences of each genetic knock-down were assessed in worms following the induction of Aβ expression under the control of a heat-inducible myocyte-specific promoter. The optimal age for Aβ induction was 48 h (stage L3) because this resulted in 50% paralysis 30 h later (Fig. S1). Induction of Aβ expression in younger worms resulted in a more rapid progression of paralysis. Rigorous primary and secondary screens identified RNAi clones that significantly (p<0.05, n = 12 with 4 biological replicates) enhanced or suppressed the Aβ-induced paralysis phenotype. Clones that modified paralysis were positively identified by DNA sequencing. In this way, we identified 78 worm genes that significantly suppressed (Table 1) the Aβ-induced paralysis phenotype. Of the 3 genes that significantly enhanced the phenotype none has a human orthologue (Table 2).

**Table 1:** Suppressors of the Aβ-paralysis phenotype.

*C. elegans* genesymbol	Human gene name	Human gene symbol	Accession number
F46E10.1	Long chain fatty acid acyl-CoA ligase	ACSF2	NM_001028767
T04C12.6	Actin and related proteins	ACTB	NM_073416
T25C8.2	Actin and related proteins	ACTG1	NM_067408
K07C5.1	Actin-related protein Arp2/3 complex, subunit Arp2	ACTR2	NM_073256
F55A12.7	AP-1 complex subunit mu-1	AP1M1	NM_059171
F29G9.3	Clathrin adaptor complex, small subunit	AP1S2	NM_072158
C13B9.3	Medium subunit of clathrin adaptor complex	ARCN1	NM_066062
C34E10.6	F0F1-type ATP synthase, beta subunit	ATP5B	NM_065710
R10E11.8	Vacuolar H+-ATPase V0 sector, subunits c/c’	ATP6V0C	NM_066764
Y49A3A.2	Vacuolar H+-ATPase V1 sector, subunit A	ATP6V1A	NM_074158
F20B6.2	Vacuolar H+-ATPase V1 sector, subunit B	ATP6V1B2	NM_076310
Y55F3AR.3	Chaperonin complex component, TCP-1 theta subunit (CCT8)	CCT8	NM_067634
F09G2.4	mRNA cleavage and polyadenylation factor II complex, subunit CFT2 (CPSF subunit)	CPSF2	NM_072421
M03F8.3	Cell cycle control protein (crooked neck)	CRNKL1	NM_001129507
B0464.1	Aspartyl-tRNA synthetase	DARS	NM_066688
C55B6.2	dsRNA-activated protein kinase inhibitor P58, contains TPR and DnaJ domains	DNAJC3	NM_076808
D2085.3	Translation initiation factor 2B, epsilon subunit (eIF-2Bepsilon/GCD6)	EIF2B5	NM_063440
F22B5.2	Translation initiation factor 3, subunit g (eIF-3g)	EIF3G	NM_001276737
C40H1.4	Elongation of very long chain fatty acids protein 3	ELOVL3	NM_066655
H19N07.1	Eukaryotic Peptide Chain Release Factor	GSPT2	NM_001269363
T10C6.11	Histone H2B	HIST1H2BA	NM_074630
F26D10.3	Molecular chaperones HSP70/HSC70, HSP70 superfamily	HSPA8	NM_070667
C49F5.1	S-adenosylmethionine synthetase	MAT1A	NM_077601
C30A5.3	MOB-Like Protein Phocein	MOB4	NM_066397
Y57G11C.12	NADH:ubiquinone oxidoreductase, NDUFA6/B14 subunit	NDUFA6	NM_070389
K07C5.4	Ribosome biogenesis protein - Nop56p/Sik1p	NOP56	NM_073259
T22B11.5	2-oxoglutarate dehydrogenase, E1 subunit	OGDHL	NM_068216
D1054.15	Isoform 1 of Pleiotropic regulator 1	PLRG1	NM_001269330
F36A4.7	RNA polymerase II, large subunit	POLR2A	NM_068122
K02B12.3	Prolactin regulatory element binding	PREB	NM_059904
R07E4.6	CAMP-dependent protein kinase type I-Alpha, regulatory subunit	PRKAR1A	NM_076598
C50C3.6	U5 snRNP spliceosome subunit	PRPF8	NM_066384
CD4.6	20S proteasome, regulatory subunit alpha type PSMA1/PRE5	PSMA1	NM_072071
Y38A8.2	20S proteasome, regulatory subunit beta type PSMB3/PUP3	PSMB3	NM_062512
C52E4.4	26S proteasome regulatory complex, ATPase RPT1	PSMC2	NM_073604
Y49E10.1	26S proteasome regulatory complex, ATPase RPT6	PSMC5	NM_067208
C23G10.4	26S proteasome regulatory complex, subunit RPN2/PSMD1	PSMD1	NM_065946
C39F7.4	GTPase Rab1/YPT1, small G protein superfamily, and related GTP-binding proteins	RAB1A	NM_070996
F10B5.1	60s ribosomal protein L10	RPL10L	NM_063306
T22F3.4	60S ribosomal protein L11	RPL11	NM_071607
C27A2.2	60S ribosomal protein L22	RPL22	NM_062531
C09H10.2	60S ribosomal protein L44	RPL36AL	NM_063974
B0250.1	60s ribosomal protein L2/L8	RPL8	NM_075539
R13A5.8	60S ribosomal protein L9	RPL9	NM_066259
F54E7.2	40S ribosomal protein S12	RPS12	NM_065820
C16A3.9	40S ribosomal protein S13	RPS13	NM_065992
T01C3.6	40S ribosomal protein S16	RPS16	NM_074289
T05F1.3	40S ribosomal protein S19	RPS19	NM_060154
C23G10.3	40S ribosomal protein S3	RPS3	NM_065948
B0393.1	40S ribosomal protein SA (P40)/Laminin receptor 1	RPSA	NM_065577
F53E10.6	RRP15-like protein	RRP15	NM_071312
VZK822L.1	Fatty acid desaturase	SCD	NM_001268666
F43D9.3	Sec1 family domain-containing protein 1	SCFD1	NM_001129225
Y113G7A.3	Vesicle coat complex COPII, subunit SEC23	SEC23B	NM_075476
Y57E12AL.1	Tumor differentially expressed (TDE) protein	SERINC1	NM_171479
T08A11.2	Splicing factor 3b, subunit 1	SF3B1	NM_065452
Y116A8C.42	Small nuclear ribonucleoprotein Sm D3	SNRPD3	NM_070626
ZK652.1	Small Nuclear Ribonucleoprotein Polypeptide F	SNRPF	NM_066307
T27F2.1	SNW domain-containing protein 1	SNW1	NM_073549
F55A11.2	SNARE protein SED5/Syntaxin 5	STX5	NM_073567
T05C12.7	Chaperonin complex component, TCP-1 alpha subunit (CCT1)	TCP1	NM_063321
Y116A8C.35	U2 small nuclear RNA auxillary factor 1 isoform b	U2AF1	NM_070635
C47E12.5	Ubiquitin-like modifier activating enzyme 6	UBA6	NM_001268520
*C46G7.1	–	–	NM_068504
*F28C6.7a	–	–	NM_063422
*Y105E8C.e	–	–	N/A
*K04E7.2	–	–	NM_076686
*F58G6.7	–	–	NM_069313
*ZK858.1	–	–	NM_060045
*Y41D4B.19	–	–	NM_067703
*F42D1.3	–	–	NM_078054
*F55C5.4	–	–	NM_073679
*F17C8.5	–	–	NM_065572
*C41C4.7a	–	–	NM_063303
*W09B6.1	–	–	NM_001267098
*D2024.1	–	–	NM_068750
*W06H8.8	–	–	NM_001029030
*F25B5.4	–	–	NM_171139

The list consists of the human orthologues of worm genes that, when targeted by RNAi, suppress the paralysis phenotype in Aβ-expressing worms (n = 78 genes).

**Table 2:** Enhancers of the Aβ-paralysis phenotype.

*C. elegans* gene symbol	Human gene name	Human gene symbol	Accession number
W02B8.3	–	–	NM_064514
F08E10.7	–	–	NM_075028
F35A5.3	–	–	NM_076258

The list consists of the human orthologues of worm genes that, when targeted by RNAi, enhance the paralysis phenotype in Aβ-expressing worms (n = 3 genes).

### Human orthologues of worm screen hits are not identical to GWAS genes but are network hubs

In our initial analysis, we generated a list of the human orthologues of the worm modifier genes using EnsemblCompara [17], and found that 61 out of the 78 worm genes had human counterparts. This ratio is significantly higher than one would expect by chance if one considers that there are only 7,970 worm genes that have human orthologues out of a genome of approximately 20,000 genes [18]. The p-value for this enrichment is p<10^−11^ (binomial test using probability of success = 7970/20000, number of successes = 61, number of trials = 78), indicating that the hits in the worm screen are much more likely to have human orthologues than one would expect by chance.

Using worm ontology enrichment analysis (clueGO plugin for Cytoscape) we found that a number of labels were over-represented in our screen hits, implicating genes involved in ATP synthesis, purine metabolism, protein binding and chaperone activity, and components of the translational machinery (Table 3).

**Table 3:** Gene ontology enrichment for worm screen hits.

Molecular function
Proton transporting ATP synthase activity, rotational mechanism
Proton transporting ATPase activity, rotational mechanism
**Cellular component**
Cytosolic small ribosomal subunit
Eukaryotic translation initiation factor 3 complex
Chaperonin-containing T-complex
**Biological process**
Protein binding
Structural constituent of ribosome
Purine nucleotide binding

The worm screen hits were analysed for enrichment in molecular function, cellular component and biological process ontology labels.

When we compared this list of 61 human orthologues of the worm modifier genes with the 63 genes in the GWAS white+grey zone (Table S1) we found no identity between the two gene lists. Indeed when we compared the ontology labels associated with the GWAS genes and the human orthologues of worm screen hits we found that there was no significant similarity (using WEB-based GEne SeT AnaLysis Toolkit, bioinfo.vanderbilt.edu/webgestalt/). However using the BioGrid database (v. 3.2.96) [19] we found that 6 of the human orthologues of worm screen genes coded for proteins that interact directly with the products of the GWAS white+grey zone genes.

In order to determine which, if any, of the worm screen hits interacted with the GWAS list to a statistically significant degree we supplemented the candidate gene lists with genes encoding the first-degree physical interactors (+1 interactome) of the protein products of the primary members using the BioGrid database (v. 3.2.96). We then asked, for each member of the worm-screen hit list, whether their +1 interactome overlap with GWAS was greater, or less, that one would expect by chance (Fig. S2). Of the 61 worm genes, 60 were present in the Biogrid database and we considered these further in our analysis. Of the 63 genes in the GWAS white+grey zone, 52 were present in Biogrid database.

**Figure 1:**
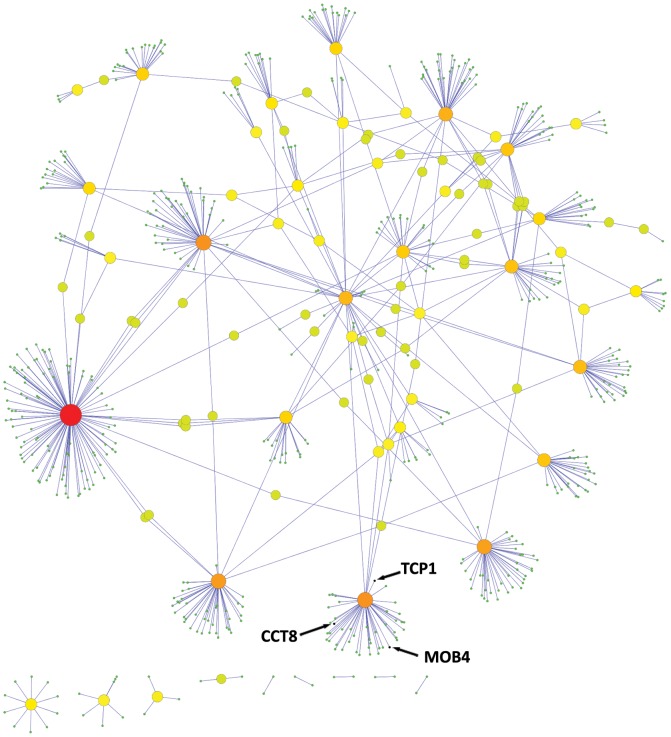
GWAS genes +1 interactome. The +1 interaction network for the 52 GWAS genes with intermediate and high significance (p<10^5^). For the 52 genes in the GWAS white+grey zones, there were 703 interactions in the +1 interactome.

**Figure 2:**
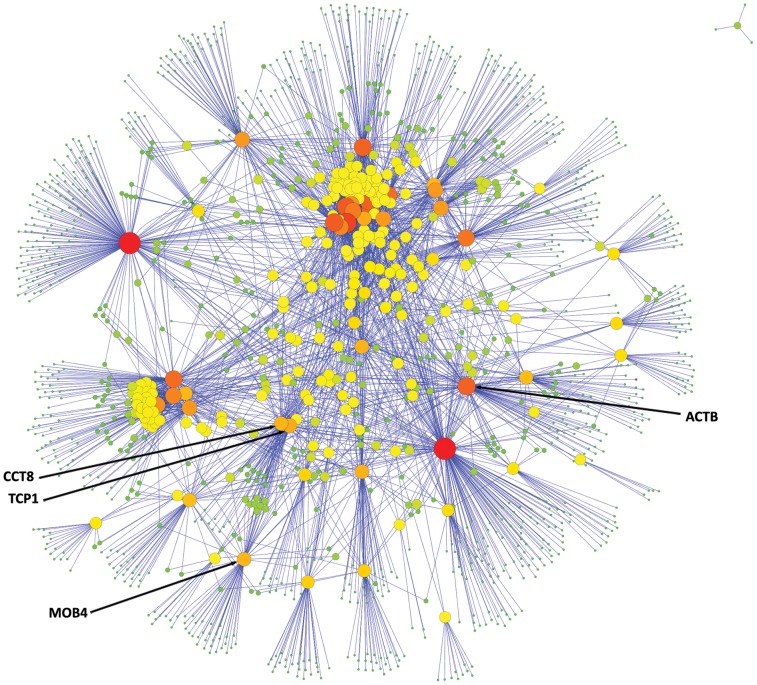
Worm genes +1 interactome. The +1 interaction network for the human orthologues of the 60 worm genes that were highlighted in our worm RNAi screen. For the 60 worm-screen genes with human orthologues, there were 3191 interactions in the +1 interactome.

It was immediately apparent from the relative sizes of the +1 interactomes (Figs. 1 & 2) that the genes derived from the worm screen were more highly interacting than the GWAS list. Specifically each worm screen gene orthologue had, on average, 53 interactors as compared to the GWAS list where the value was less than 14.

### Ranking the distribution of gene interconnectivity

In order to determine whether the observed bias towards highly interconnected genes in the worm modifier list was statistically significant, we used a computational approach to compare our observed population of genes to similar, randomly generated, lists. Firstly we ranked the population of human genes in the Biogrid database (13940 genes) according to their number of interactors. This ranked list was then divided into 10 bins such that each bin contained the same number of human orthologues of worm genes (that is 797 in each bin). For example, this resulted in the most highly interconnected 952 human genes in the database being put into the first bin; the bin boundaries for the whole database were: 1–952, 953–2006, 2007–3175, 3176–4403, 4404–5735, 5736–7134, 7135–8624, 8624–10200, 10201–12104 and 12105–13928. Using a Monte-Carlo approach [20] we then generated 100 lists containing 60 randomly generated human genes with worm orthologues.

**Figure 3:**
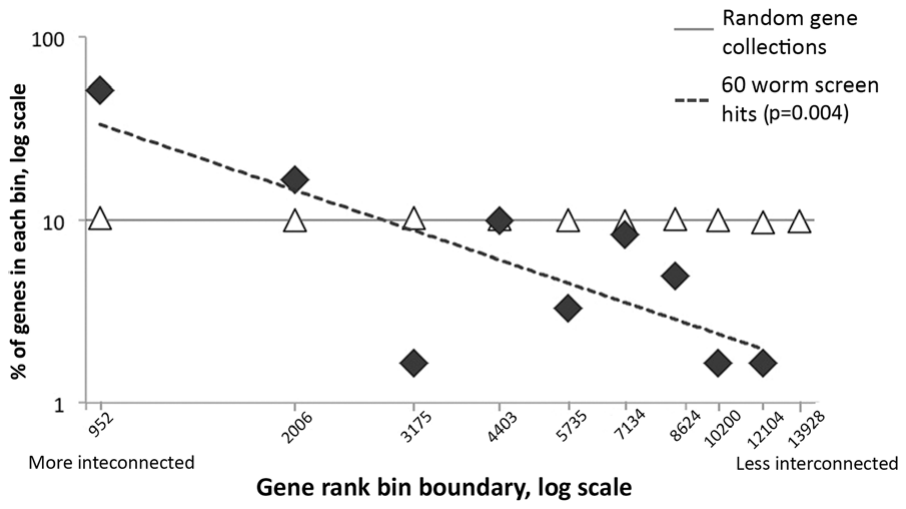
Distribution of rankings of worm genes. The x-axis represents the log of the gene ranking bin boundaries, arranged in decreasing gene-product connectedness from left to right. The y-axis represents the log of the fraction of genes in each bin (where 100% is 60 genes). The dashed line shows the linear regression for the worm screen results. The results of the screens are shown as black diamonds and results of random simulations are shown as empty triangles.

As expected the genes in the random lists segregated with equal frequency into the various bins (Fig. 3, triangles); however, a consequence of the highly interconnected nature of the worm screen gene list is that >50% of its members (31 out of 60) fall within the most highly interconnected bin on the left side of graph. More generally, the worm screen gene list exhibited a power law distribution such that we could fit a linear regression line (gradient −1.1) to a log-log plot of gene rank bin boundary vs. frequency of observation (Fig. 3, diamonds).

To determine whether the bias is statistically significant we performed a two-tailed Student’s t-test [21] to see whether the gradient of the experimental regression line is different from the data generated in the Monte Carlo simulations. A p-value of 0.004 indicates that indeed the hits from our worm screen are more highly interconnected that a comparable random population.

**Figure 4:**
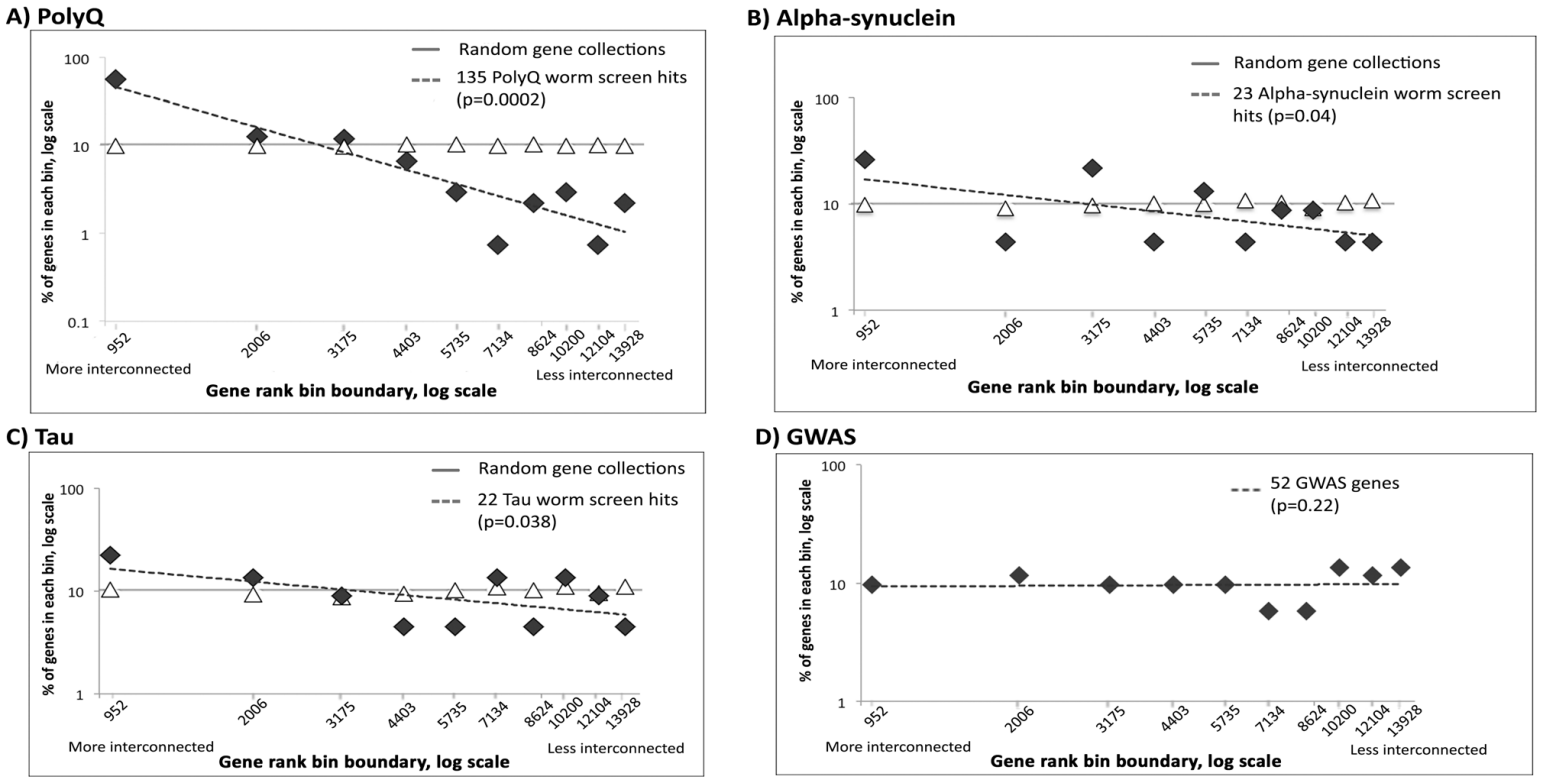
Distribution of rankings of worm models of neurodegenerative diseases. The x-axis represents the log of the gene ranking bin boundaries, arranged in decreasing gene-product connectedness from left to right. The y-axis represents the log of the fraction of genes in each bin (where 100% is 135, 23, 22 & 52 genes for panels A, B, C & D respectively). The dashed line shows the linear regression for the worm screen results. The results of the screens are shown as black diamonds and results of random simulations are shown as empty triangles. (A) polyglutamine screen; (B) α-synuclein screen; (C) tau screen; (D) GWAS candidate white+grey zone genes for AD.

To understand whether such a bias is a common feature of worm RNAi screens, we repeated a similar analysis for three published modifier screens of human neurodegenerative disorders (modelling polyglutamine [22] [Table S2a], tau [23] Table S2b] and alpha-synuclein pathogenesis [24] [Table S2c]) and, congruent with our results, we also found a skew in these screens towards highly interconnected genes (Fig. 4 A–C). Using a one-tailed Student’s t-test we observed that for the polyQ (p = 0.0002, Fig. 4, panel A), the α-synuclein (p = 0.04, panel B), and the tau (p = 0.04, panel C) screens, the gradient of the linear regression lines was significantly less than for the Monte-Carlo-generated data. By contrast the equivalent gradient of the GWAS white+grey zone genes was robustly zero (p = 0.22, two-tailed, panel D).

The bias toward highly interacting networks in the results of a number of worm modifier screens, including our own, precludes any simple analysis of the overlap between the worm screen and GWAS +1 interactomes. This is because some of the worm genes have several hundred interactors and are likely to generate an overlap by chance. For example HSPA8 constitutes over 30% of the overlap using the hits from our Aβ screen. For these reasons, we developed a more robust method for determining whether the overlap in the worm and GWAS interactomes was significant or not.

### Determining the statistical significance of the overlap between worm screen hits and GWAS results

To compensate for the bias towards highly interacting genes in the worm screen, we designed random Monte Carlo simulations in which each simulated worm +1 interactome has the same size as the particular worm screen gene product being tested.

The statistical significance (p-value) of the overlap between the +1 interactome of each worm screen hit and the GWAS +1 interactome was achieved by generating 1000 random worm +1 interactomes of equivalent size for each experimental worm gene. We then counted how often the overlap of the random worm +1 interactomes with the GWAS +1 interactome was either> = or< = to the experimentally observed overlap. To quantify the statistical significance of our findings, we repeated this process for 100 random lists of 60 worm genes.

Overall, we found that, of the 100 random lists of 60 worm genes, 3.14 (95% confidence interval = ±0.34) genes had +1 interactome overlaps with GWAS that differed significantly from expected at a level of p<0.05. As such, this number is consistent with the random nature of the simulated gene lists. However when we examined the experimental worm screen list, we found that 7 genes had a non-random +1 interactome overlap with the GWAS network. When we reviewed our simulations we found that out of 100 runs only 5 resulted in 7 or more genes that had a +1 interactome overlap that differed from chance, yielding p = 0.05 for our observation.

### Genes with non-random +1 interactome overlaps

**Figure 5:**
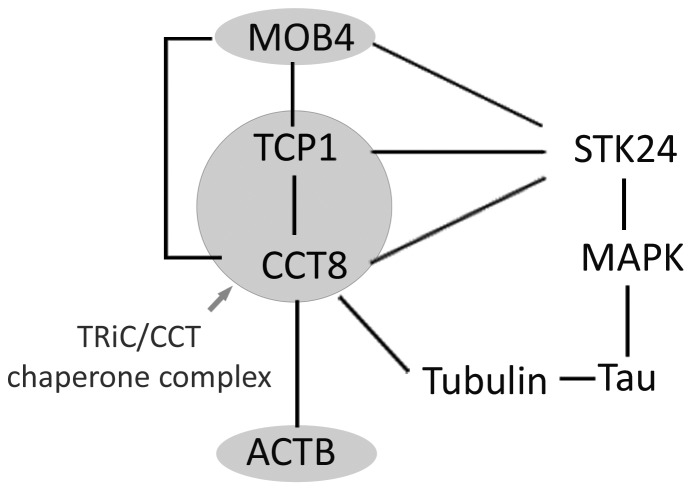
The interaction network of 4 significant worm gene products. Four human orthologues of worm screen gene products (MOB4, TCP1, CCT8 and ACTB) have +1 interactomes that overlap more than expected with the +1 interactome of the GWAS white+grey gene products. Two of these are components of the abundant cytoplasmic TRiC/CCT chaperone, the third (MOB4) interacts closely and fourth (ACTB), along with tubulin, is an important substrate. TRiC/CCT interacts with STK24 and PP2a to form a complex that regulates the MAPK pathway. This network of interactions may have a bearing on tau phosphorylation and neuronal regeneration.

When we considered the overlap between the 7 non-random worm genes we found that 3 (NOP56, PSMA1 and RPL10L; Fig. S5) had a lower +1 interactome overlap with the GWAS list than would be expected by chance. The remaining four worm genes are of particular interest because they have a larger +1 interactome overlap with GWAS than expected. Remarkably, two of these genes (TCP1, CCT8) are components of the TRiC/CCT chaperone complex and the third (MOB4) interacts closely [25], [26]. The fourth gene is one of the major folding substrates of the chaperone, namely actin (ACTB). Furthermore, TCP1, CCT8 and MOB4 all interact directly with a single GWAS grey zone gene, namely the kinase STK24 [26] (Fig. 5).

## Discussion

Genome-wide association studies [1], [13], [14] and a subsequent targeted genotyping screen [15] have shown that none of the genes implicated in FAD carry common variants that confer risk for the sporadic version of the disease. These results have called into question the validity of using familial AD, where the pathogenesis is undoubtedly related to Aβ generation, as a model for the more common sporadic AD. In particular, it is possible that the sporadic disease may have a distinct pathogenesis, perhaps based on immune dysregulation, that is not based on aberrant APP metabolism and the consequent production of Aβ. To address this issue, we have employed an invertebrate model system that exclusively reports the toxicity of Aβ; that is, a worm expressing Aβ under the control of a muscle-specific driver. While, in this particular model system, the paralysis is likely to be caused by muscle damage, rather than neurodegeneration, it is known that Aβ toxicity can be observed across a wide range of cell types and the mechanisms may well be conserved.

The worm screen in this study has reported on worm gene transcripts that, when targeted by RNAi, modify the Aβ-induced paralysis phenotype. The deployment of *rol-6* as a marker of Aβ expression means that we can rule out any marked artefactual loss of the expression array in response to RNAi. However, we did not measure expression levels of the Aβ peptide in response to each RNAi treatment, so we cannot exclude the possibility that for a particular RNAi treatment that effects on paralysis could be attributed to changes in transgene expression rather than an effect on Aβ toxicity. Furthermore this study can usefully be extended by including worm lines carrying mutant alleles of the genes implicated by the RNAi studies both in mammalian systems and in *C. elegans*.

In this study our goal was to use the results of the worm genetic screen to detect GWAS genes with borderline significance that were nevertheless involved in the pathogenesis of sporadic AD. Our initial, and most stringent, test indicated that none of the genes in the GWAS white or grey zones were identical to the human orthologues of the 61 worm modifier genes. This negative finding suggests that, at least for the predominantly Caucasian populations in which GWAS studies have been performed, Aβ toxicity cannot be considered as identical to sporadic AD. Rather the data support the idea that other pathological pathways may be important in the disorder. However, we did observe that 6 worm gene orthologues were predicted to interact physically with members of the GWAS white+grey zone gene products (Table 4, asterisks). It was also notable that worm modifier genes were much more likely than chance to have a human orthologue. This involvement of a core and essential and highly conserved genes reflects the profound toxic effect posed by Aβ.

**Table 4:** Overlap between the +1 interactome for the products of genes in the GWAS white+grey zone and those of genes in the +1 interactome of the worm RNAi screen.

Gene	p-value	+1 interactome overlap [experimental vs MC]
**MOB4**	**0.003**	larger*
**PSMA1**	**0.014**	**smaller**
**TCP1**	**0.014**	larger*
**NOP56**	**0.014**	**smaller**
**CCT8**	**0.015**	larger*
**ACTB**	**0.018**	**larger**
**RPL10L**	**0.033**	**smaller**
RPL9	0.058	smaller
ATP6V1B2	0.064	smaller
HSPA8	0.065	larger
RPL22	0.087	smaller
PSMB3	0.089	smaller
RPL11	0.097	smaller
RPS13	0.12	smaller
PSMC2	0.131	smaller
PSMD1	0.137	smaller
PRPF8	0.141	smaller*
ACTR2	0.153	larger
ACTG1	0.159	larger*
PLRG1	0.168	smaller
SCFD1	0.189	smaller
AP1M1	0.196	smaller
RRP15	0.199	larger
U2AF1	0.199	smaller
RPS12	0.233	smaller
SNW1	0.253	larger
RPSA	0.272	smaller
ARCN1	0.289	smaller
SCD	0.289	larger
ATP5B	0.299	larger
RPS19	0.31	smaller
RPS16	0.313	smaller
DARS	0.314	smaller
RPL8	0.322	smaller
STX5	0.33	smaller
POLR2A	0.331	smaller
GSPT2	0.333	larger
EIF3G	0.334	larger
DNAJC3	0.343	smaller
PSMC5	0.346	smaller*
CPSF2	0.362	smaller
SEC23B	0.371	larger
RAB1A	0.382	smaller
SF3B1	0.421	smaller
PRKAR1A	0.435	smaller
RPS3	0.437	smaller
OGDHL	0.449	larger
SNRPD3	0.458	smaller
ACSF2	0.467	larger
NDUFA6	0.486	larger
UBA6	0.515	larger
CRNKL1	0.535	larger
MAT1A	0.539	smaller
RPL36AL	0.55	larger
ATP6V1A	0.557	larger
SERINC1	0.577	larger
EIF2B5	0.58	smaller
HIST1H2BA	0.58	smaller
AP1S2	0.655	smaller
ATP6V0C	0.759	larger

The human AD GWAS white+grey zone genes, and their +1 interactors, overlap with the human orthologues of worm-screen hits, and their +1 interactors. Worm screen genes that have human orthologues with a significantly non-random +1 interactome overlap with the GWAS list are shown in bold. Worm screen genes that have human orthologues that interact directly with GWAS genes are marked with an asterisk.

In order to determine whether the degree of interaction between the worm and GWAS genes was greater, or less, than one would expect by chance we systematically compared the overlap between the worm and GWAS +1 interactomes for each of the worm-screen genes (Fig. S2).

### Worm-screen hits, but not the GWAS list, are highly interconnected genes

Considering the +1 interactomes of the worm genes from our Aβ-based screen, we observed that there was a clear bias toward genes encoding highly interactive proteins. As can be seen from Fig. 3, there is a power law relationship between the frequency with which we observe particular gene products and their particular degree of interconnectedness. For our screen, the gradient of the linear fit of the log-log plot is −1.1. This is significantly different from a gradient of zero, which we observed for a random set of genes where the chance of observing a gene is independent of its degree of interconnectivity.

We were interested to know whether the gene lists from other genetic screens in worm models of human disease also generated similarly skewed distributions of connectivity. Indeed, when we analysed the data for polyQ, synuclein and tau screens, we found that they all had a negative slope (−1.4, −0.5, and –0.4, respectively) and all were significantly different from the expected zero value.

One possible explanation for the highly interconnected nature of the worm screen hits could be summarised as “sociological bias” [27]. In this context, we hypothesise that previous worm screens have yielded similar gene lists and that the consequent interest in the field has resulted in more interaction data being deposited in the BioGrid database. In support of this hypothesis, we find that the screens for Aβ and polyQ yield 27 common genes, and we find that 20 of them are highly interconnected genes (present in the first bin in plots in Figs. 3 & 4). Even if there is such a sociological bias, this does not invalidate subsequent conclusion because we have carefully compensated for differences in network connectivity.

Another explanation is that genetic modifiers of worm phenotypes really do have more highly interconnected protein products as compared to the average gene [28]. Highly interconnected gene products are likely involved in more biological pathways than less connected ones; so it may be that highly interconnected gene products are more likely to influence disease-related processes as assessed by a genetic screen. However this cannot explain why there is no similar interconnectivity bias when the same analysis is applied to the GWAS white+grey zone genes. Indeed, the distribution of connectivity of the products of white+grey zone genes is remarkably similar to that of the general population of genes. This is likely to be explained by the presence of many false-positive genes in this list.

### Worm-GWAS overlap analysis implicates the TRiC/CCT chaperone in mediating Aβ toxicity in sporadic Alzheimer’s disease

For each gene in the worm-screen hit list we estimated whether its interactome overlapped with the GWAS interactome to a greater or lesser extent than would be expected by chance. We employed a Monte Carlo simulation approach where we generated 1000 random interactomes that were the same size as the experimental interactome (Fig. S3) and determined the distribution of the overlap scores (Fig. S4). For 7 of the 60 worm genes we found that the overlap differed significantly from the chance expectation.

Three of these genes (NOP56, PSMA1 and RPL10L) have +1 interactome overlaps with GWAS white+grey zone genes that are smaller than one would expect by chance, suggesting that they are artifactual modifiers of the worm phenotype and unlikely to be involved in the human disorder. Consequently, these data should dissuade us from investigating the ribosomal [29] and proteasomal [30] functions of these genes in AD. By contrast, the remaining 4 genes exhibited a greater than expected +1 interactome overlap with the GWAS white+grey zone genes. Furthermore, we found that two of these genes (TCP1, CCT8) are both members of the TRiC/CCT chaperone that has a number of substrates including actin (ACTB is the fourth gene) and tubulin. This chaperone complex was also enriched in the gene ontology analysis of the worm hits (Table 3). CCT8 and MOB4 have not been observed as modifiers in any other worm models of neurodegeneration however TCP1 and ACTB RNAi also modify polyQ toxicity [22], [23], [24]. While this may represent conservation of pathological mechanisms between Aβ and polyQ it may alternatively indicate that TCP1 and ACTB RNAi constructs have non-specific effects in worm model systems. It is notable that TCP1 levels are abnormal in foetal Down’s syndrome [31]. Furthermore TCP1, CCT8 and MOB4 all interact with each other and also with the kinase STK24, a member of the GWAS grey zone, that has been linked to axonal regeneration in rats [32]. These three candidate genes, along with STK24 and PP2A, form a multiprotein complex that may regulate the MAPK pathway [33]. From our analysis, we propose that both neuroregenerative pathways linked to STK24 and also the MAPK-linked phosphorylation of proteins such as tau [34] may be the mechanisms by which the TRiC/CCT chaperone exerts its effects in AD.

The details of the role for TRiC/CCT in AD are unknown; however the finding that RNAi knockdown in the worm protects against Aβ-induced paralysis indicates that the chaperone in some way mediates Aβ toxicity. TRiC has been shown to remodel oligomeric protein aggregates in a worm model of polyQ toxicity, with a concomitant reduction in toxicity [35]. It is therefore surprising that knock down TRiC components is protective in our model of Aβ toxicity, however there are a number of precedents for a reduction of chaperone activity having a protective effect (see [36]–[42]) and it is hypothesised that low levels of misfolded proteins may induce, or help maintain, the activity of regenerative pathways. Our additional finding that actin RNAi is also protective in the worm screen may indicate that reducing the levels of this important substrate for TRiC/CCT promotes the ability of the chaperone to interact with STK24 and so promote neuroregeneration.

## Conclusions

In summary, the lack of a direct overlap between genes implicated by the GWAS studies and the hits from our modifier screen in *C. elegans* indicates that AD cannot simply be considered synonymous with Aβ toxicity. However using a +1 interactome network approach we have shown that the experimental pattern of overlap between worm and GWAS gene products was significantly non-random. Of those worm genes that showed increased overlap with GWAS lists, three are part of an abundant cytoplasmic complex with important chaperone activity; the fourth gene coded for β-actin that is an important substrate of the same chaperone. The interaction that the TRiC/CCT chaperone has with tubulin, STK24 and the MAPK pathway may explain how Aβ promotes tau-linked cytoplasmic pathology.

## Methods

### *C. elegans* strains

The *C. elegans* strain CL4176 [*smg-1*(cc546ts) dvIs27(pAF29 Pmyo-3::Aβ42+pRF4)], expressing Aβ in body-wall muscle, and the non-Aβ expressing control, CL802 [*smg-1*(cc546ts) *rol-6*(su1006)] have been described previously [43]. The worms were routinely cultured at the permissive temperature of 16°C on solid peptone nematode growth media (NGM) as described previously [44].

### RNA interference (RNAi) treatment of *C. elegans*

Clones of HT115 *E. coli* carrying RNAi expression plasmids from the whole-genome *C. elegans* feeding library (Geneservice, UK) were cultured separately, in 500 µl LB medium containing 50 µg/ml ampicillin, overnight at 37°C. A control culture, carrying empty plasmids without an RNAi insert, was grown simultaneously under identical conditions. Bacteria were seeded drop-wise onto solid NGM plates, supplemented with 25 µg/ml carbencillin and 1 mM IPTG, and incubated overnight at room temperature. Worm embryos were harvested from synchronised worms as described previously [45] and transferred to NGM agar plates seeded with RNAi-expressing, or control, bacteria and left 48 h to develop into L3 larvae. Aβ expression was then induced by increasing the ambient temperature to 23°C for 24 h, when the worms were scored for the incidence of the paralysis phenotype.

### Primary screen to detect RNAi modifiers of Aβ-induced paralysis

The interaction of each RNAi-expressing clone with the Aβ paralysis phenotype in the worm was assessed in triplicate and compared with triplicate wells of worms fed with control bacteria carrying an empty vector. Each plate also had a well containing bacteria expressing RNAi to *unc-22* that normally results in uncoordinated body wall twitching. As a quality control, any plate in which the *unc-22* RNAi-fed worms failed to twitch was discarded and the experiment was repeated.

Locomotor behaviour was assessed at two time-points following the increase in ambient temperature. The first assay was performed 22 h post-induction, at which time Aβ-expressing worms fed control bacterial cultures were all moving normally. When all worms in each of the triplicate wells for a particular RNAi clone at this time point were seen to be paralysed, then the clone was defined as an enhancer of Aβ toxicity. This enhancement was specific to Aβ-expressing worms as no similar paralysis was seen in the corresponding treatment of non-Aβ-expressing CL802 worms. Clones that non-specifically paralyse worms were not considered. The second time-point, at 32 hr, permitted the detection of suppressors of the paralysis phenotype. At this time-point, Aβ-expressing worms under control conditions were all paralysed and so any RNAi-expressing clone that protected the locomotor function of all the worms, in triplicate wells, was defined as a suppressor of Aβ toxicity.

### Secondary screen to confirm the RNAi modifiers of Aβ-induced paralysis

The secondary screen consisted of a further three experiments, each performed in triplicate wells, for each hit from the primary screen. Paralysis curves were generated by hourly observation of locomotor behaviour and were analysed using Kaplan-Meier plots with statistical significance being estimated using the Mantel-Cox Log Rank test (GraphPad, Prism); p<0.05 was considered significant.

### Finding human orthologues of worm genes

According to our pre-planned study design, we considered only those genes in *C.elegans* that have one or more human orthologues. We used the EnsemblCompara phylogenetically based orthology prediction method (http://www.ensembl.org/) [17] to find human orthologues of worm genes with minimum 25% amino acid identity [46] with respect to the query gene product. In cases where there was more than one human orthologue, then the gene with the highest % identity was chosen. In approximately 15% of cases one worm gene had multiple human orthologues all with the same percent identity; in these cases, the preferred orthologue was chosen using MetaPhOrs (http://orthology.phylomedb.org/) [47] where the consistency score for any orthology was used as the tie-breaker. This score is 1 where an orthology is supported by all the available databases and decreases as agreement is less consistent.

Using this method, human orthologues for 61 out of 78 worm genes were found. The human orthologues of this series of worm genes was then compared with the GWAS white+grey zone genes (Fig. S2.1) as published at the Database of Genotype and Phenotype (dbGaP) of the National Center for Biotechnology Information [48] (http://www.ncbi.nlm.nih.gov/projects/gapplusprev/sgap_plus.htm, p<10^−5^, Disease = “Alzheimer Disease”, access date June 2013).

### Network Generation

The “GWAS +1 interactome” consists of all the GWAS white+grey zone genes plus all the genes encoding proteins with which their products physically interact. The “worm +1 interactome” was generated in a similar manner, using 61 human orthologues of the worm modifier screen “hits”. The list of interactions was retrieved from the “Biogrid” database (http://thebiogrid.org, v. 3.2.96) [19]. The statistical significance of the overlap between the worm +1 interactome and the GWAS +1 interactome was determined by Monte Carlo simulation [49].

### Monte Carlo simulations

To test whether any particular worm screen hit *g* had a +1 interactome that overlapped more with the GWAS +1 interactome than expected by chance, we used the following computational algorithm (Fig. S2):

1. The size of the +1 interactome for each human orthologue of the worm screen hits was determined.
2. Random +1 interactomes, of the same size as the network in step 1, were then generated (Fig. S3). This was achieved by randomly choosing one of the 7970 human orthologues of worm genes, and then generating its +1 interactome and using this list to progressively populate a random +1 interactome. Once this random list had reached the required length, the process was terminated. If, however, the first randomly selected human orthologue of a worm gene had insufficient interactors to completely populate the required random +1 interactome, then additional random genes and their associated interactor lists were added until the random list was complete.
3. The generated set of random genes was compared with the +1 interactome of the GWAS white+grey zone genes and the number of identical genes was counted (the overlap).
4. Steps 2–3 were repeated 1000 times, yielding a Monte Carlo set of 1000 overlap scores.
5. The number of times that the random overlap was equal to, or greater, than the experimental overlap was counted - this was termed n*_right,g_* (Fig. S4, panel A)
6. Similarly the number of times that the random overlap was equal, or less, than the experimental score was also counted - this was termed n*_left,g_* (Fig. S4, panel B)
7. We divided either n*_right,g_* or n*_left,g_*, whichever was smaller, by the number of simulations (n = 1000) to give the p-value for the overlap (Fig. S2), thus ensuring that a one-tailed test was simulated.

## Supporting Information

Figure S1 **Paralysis timecourse following induction of Aβ expression.** Increasing the ambient temperature to 23°C when the worms are 48 h old (stage L3) induces Aβ expression and results in progressive paralysis in worms fed on *E. coli* containing empty vector (a, round symbols). By contrast, a typical suppressor clone rescues this locomotor deficit (square symbols). The paralysed worms (b, arrows) are straighter and largely immobile whereas non-paralysed worms exhibit a marked “roller” phenotype (c, arrowheads), but otherwise move normally.
(TIF)

Figure S2 **Computational pipeline for determining the significance of the overlap between the +1 interactomes of worm-screen hits and GWAS white+grey zone genes.** The algorithm is described more fully in the “Monte Carlo simulations” section of the methods section.
(TIF)

Figure S3 **Generation of equivalent random +1 interactomes.** An illustration of how random +1 interactomes (right) were generated to compare with the +1 interactomes of each of the worm screen hits. Random +1 interactomes were generated containing the same number of genes (in this example, n = 11). Human orthologues of worm-screen hits are labeled with “1” (left). Random human genes with a worm orthologue are labeled with “2” (right). Human +1 interactors are represented by small green dots.
(TIF)

Figure S4 **Estimating the significance of highly overlapping/poorly overlapping +1 interactomes.** The +1 interactome of the human orthologues of worm screen genes may overlap more than expected (panel A, contributing to n_right_) or less than expected (panel B, contributing to n_left_). Generation of 1000 random +1 interactomes allows the p value for the experimental +1 interactome to be derived. Further details can be found in the “Monte Carlo simulations” section of the methods section.
(TIF)

Figure S5 **Venn diagram indicating the degree of the +1 interactome overlap for GWAS white+grey genes and each of the significant genes from the worm screen.** Seven genes from the worm screen have human orthologues that have +1 interactomes that overlap more or less than one would expect by chance with the GWAS +1 interactome. Four overlap more (left panel), and three less (right panel), than expected. The area of each circle or overlap is proportional to the number of genes. Worm screen genes that have human orthologues that interact directly with GWAS white+grey zone gene products are marked with asterisks.
(TIF)

Table S1 The 63 human genes in the AD GWAS white+grey zones.
(DOCX)

Table S2 Worm RNAi modifiers for screens of various disease models. The genes that modified fly phenotypes when targeted with RNAi are listed for a) polyglutamine, b) tau and c) α-synuclein.
(DOCX)
