# Supplementary figures and images for: The TRiC/CCT Chaperone Is Implicated in Alzheimer's Disease Based on Patient GWAS and an RNAi Screen in Aβ-Expressing Caenorhabditis elegans
Source: PLoS One. 2014 Jul 31;9(7):e102985. doi: 10.1371/journal.pone.0102985 (PMC4117641; doi:10.1371/journal.pone.0102985)

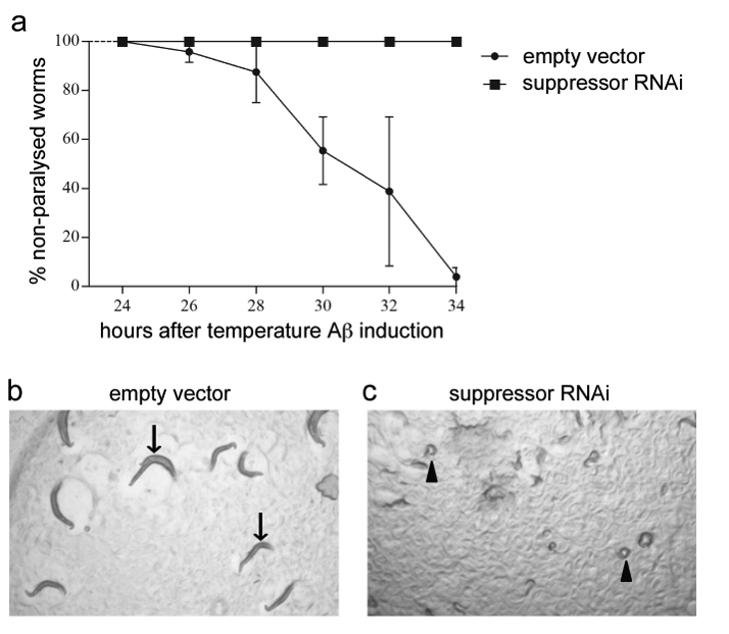

Supplement: Figure S1 — Paralysis timecourse following induction of Aβ expression. Increasing the ambient temperature to 23°C when the worms are 48 h old (stage L3) induces Aβ expression and results in progressive paralysis in worms fed on E. coli containing empty vector (a, round symbols). By contrast, a typical suppressor clone rescues this locomotor deficit (square symbols). The paralysed worms (b, arrows) are straighter and largely immobile whereas non-paralysed worms exhibit a marked “roller” phenotype (c, arrowheads), but otherwise move normally. (TIF) [file pone.0102985.s001.tif]

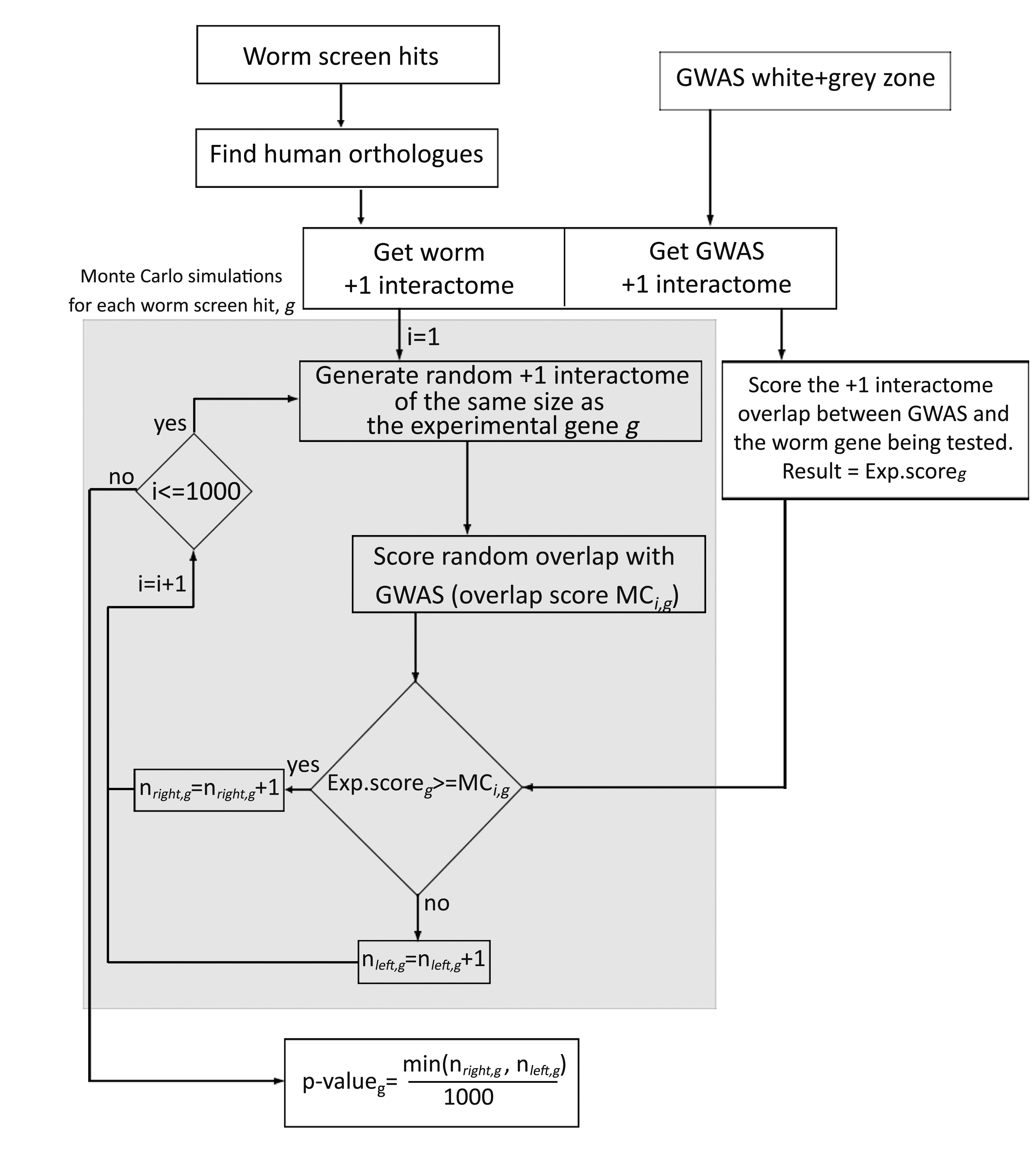

Supplement: Figure S2 — Computational pipeline for determining the significance of the overlap between the +1 interactomes of worm-screen hits and GWAS white+grey zone genes. The algorithm is described more fully in the “Monte Carlo simulations” section of the methods section. (TIF) [file pone.0102985.s002.tif]

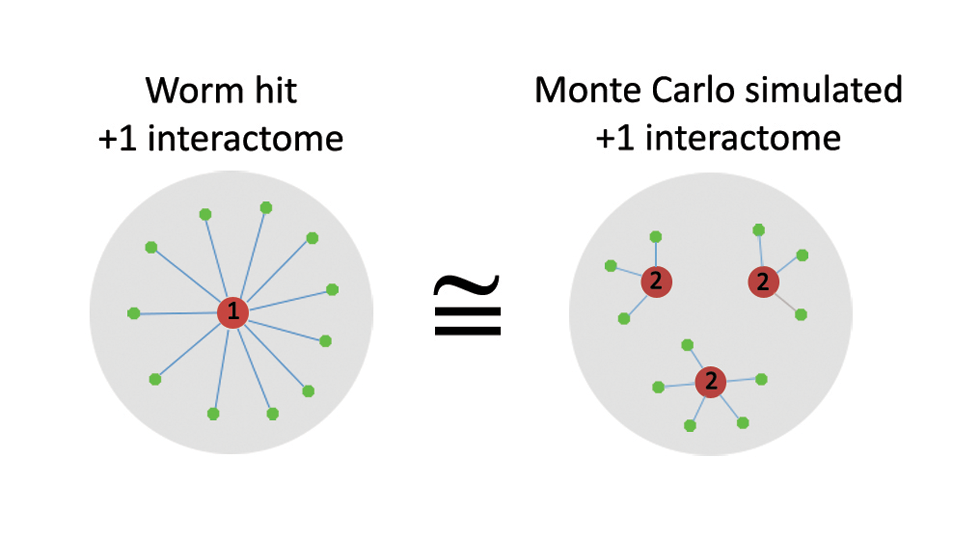

Supplement: Figure S3 — Generation of equivalent random +1 interactomes. An illustration of how random +1 interactomes (right) were generated to compare with the +1 interactomes of each of the worm screen hits. Random +1 interactomes were generated containing the same number of genes (in this example, n = 11). Human orthologues of worm-screen hits are labeled with “1” (left). Random human genes with a worm orthologue are labeled with “2” (right). Human +1 interactors are represented by small green dots. (TIF) [file pone.0102985.s003.tif]

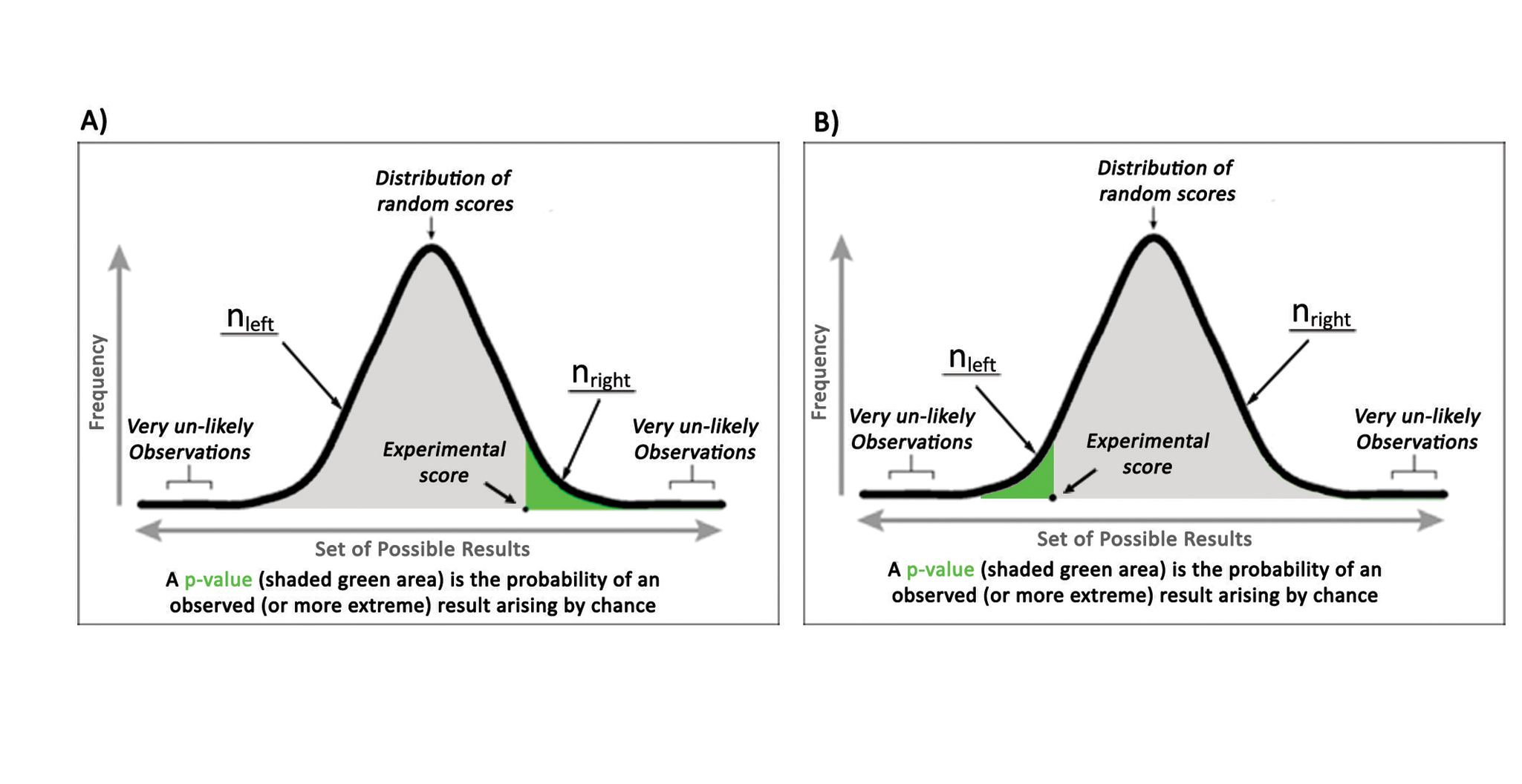

Supplement: Figure S4 — Estimating the significance of highly overlapping/poorly overlapping +1 interactomes. The +1 interactome of the human orthologues of worm screen genes may overlap more than expected (panel A, contributing to nright) or less than expected (panel B, contributing to nleft). Generation of 1000 random +1 interactomes allows the p value for the experimental +1 interactome to be derived. Further details can be found in the “Monte Carlo simulations” section of the methods section. (TIF) [file pone.0102985.s004.tif]

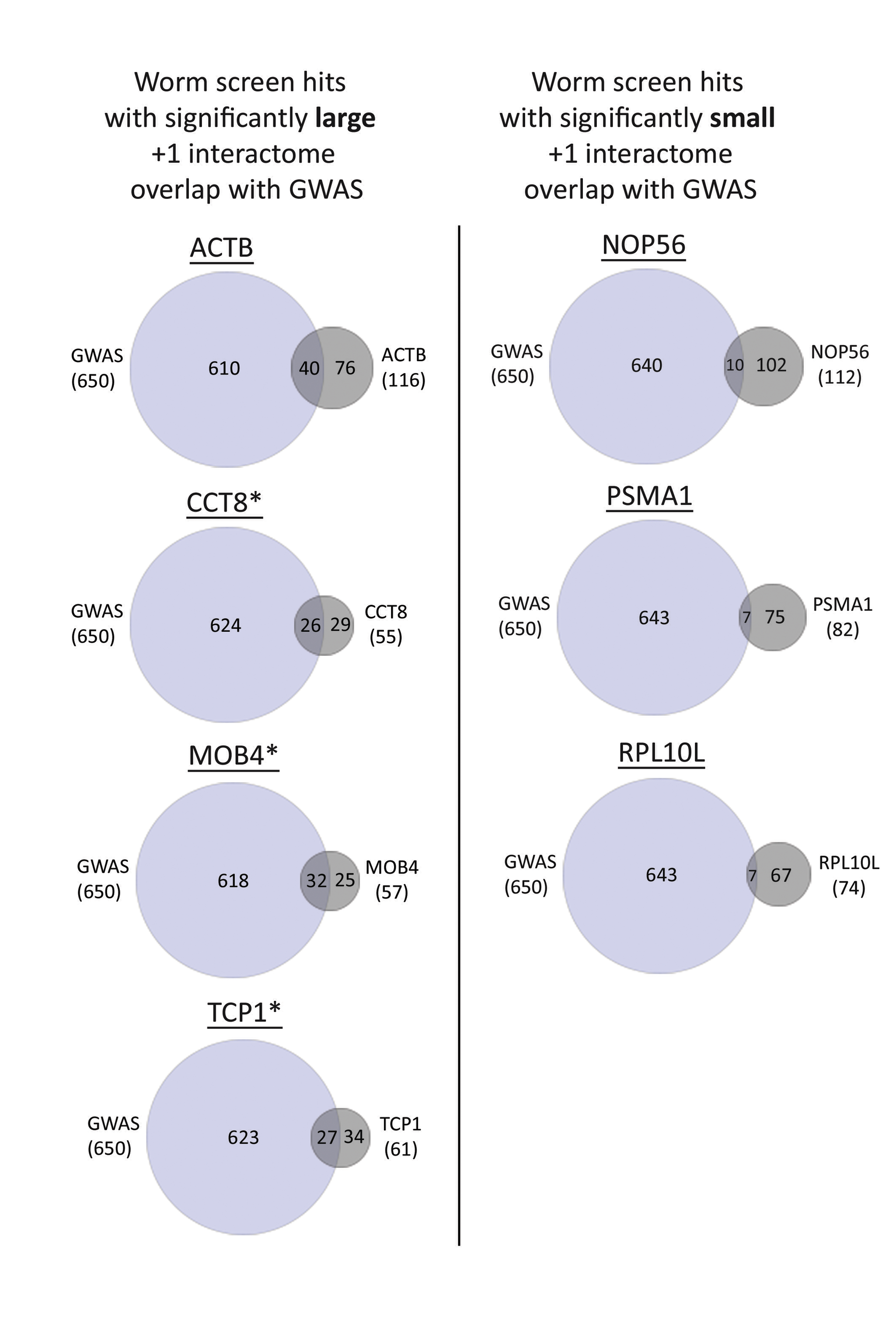

Supplement: Figure S5 — Venn diagram indicating the degree of the +1 interactome overlap for GWAS white+grey genes and each of the significant genes from the worm screen. Seven genes from the worm screen have human orthologues that have +1 interactomes that overlap more or less than one would expect by chance with the GWAS +1 interactome. Four overlap more (left panel), and three less (right panel), than expected. The area of each circle or overlap is proportional to the number of genes. Worm screen genes that have human orthologues that interact directly with GWAS white+grey zone gene products are marked with asterisks. (TIF) [file pone.0102985.s005.tif]
