# Supplementary material for: The TRiC/CCT Chaperone Is Implicated in Alzheimer's Disease Based on Patient GWAS and an RNAi Screen in Aβ-Expressing Caenorhabditis elegans
Source: PLoS One. 2014 Jul 31;9(7):e102985. doi: 10.1371/journal.pone.0102985 (PMC4117641; doi:10.1371/journal.pone.0102985)
Supplement: Table S1 — The 63 human genes in the AD GWAS white+grey zones. (DOCX) [file pone.0102985.s006.docx]

**Table S1: The 63 human genes in the AD GWAS white+grey zones.**

| 1 | ABCA1 |
| --- | --- |
| 2 | ABCA7 |
| 3 | ADCY8 |
| 4 | AEN |
| 5 | ANK1 |
| 6 | APOC1 |
| 7 | APOE |
| 8 | BIN1 |
| 9 | CCDC134 |
| 10 | CCDC76 |
| 11 | CD2AP |
| 12 | CD33 |
| 13 | CLU |
| 14 | CLVS1 |
| 15 | CNTNAP2 |
| 16 | CR1 |
| 17 | CUBN |
| 18 | CYP7B1 |
| 19 | DBT |
| 20 | DCHS2 |
| 21 | DIAPH3 |
| 22 | DIP2C |
| 23 | DISC1 |
| 24 | DNAH11 |
| 25 | EML1 |
| 26 | ENSAP3 |
| 27 | F13A1 |
| 28 | FARP1 |
| 29 | GAB2 |
| 30 | GFRA2 |
| 31 | GRIN2B |
| 32 | LRRC39 |
| 33 | MGST3 |
| 34 | MPP7 |
| 35 | MS4A6A |
| 36 | MTHFD1L |
| 37 | NCAM1 |
| 38 | NCR2 |
| 39 | NDP |
| 40 | NEDD9 |
| 41 | NHSL1 |
| 42 | ODZ4 |
| 43 | PAK2 |
| 44 | PCDH11X |
| 45 | PITPNC1 |
| 46 | POLN |
| 47 | PVRL2 |
| 48 | RBFOX1 |
| 49 | RORA |
| 50 | RYR2 |
| 51 | SLC28A1 |
| 52 | SLC4A1AP |
| 53 | SLC44A5 |
| 54 | ST3GAL1 |
| 55 | STK11 |
| 56 | STK24 |
| 57 | TOMM40 |
| 58 | TXNDC6 |
| 59 | UTS2D |
| 60 | VSNL1 |
| 61 | WBSCR17 |
| 62 | ZNF292 |
| 63 | ZNF320 |
