# Supplementary material for: The TRiC/CCT Chaperone Is Implicated in Alzheimer's Disease Based on Patient GWAS and an RNAi Screen in Aβ-Expressing Caenorhabditis elegans
Source: PLoS One. 2014 Jul 31;9(7):e102985. doi: 10.1371/journal.pone.0102985 (PMC4117641; doi:10.1371/journal.pone.0102985)
Supplement: Table S2 — Worm RNAi modifiers for screens of various disease models. The genes that modified fly phenotypes when targeted with RNAi are listed for a) polyglutamine, b) tau and c) α-synuclein. (DOCX) [file pone.0102985.s007.docx]

**Table S2: Worm RNAi screen hits for various modifier screens**

a) Worm RNAi screen genes modelling polyglutamine disease

| **Worm gene** | **Human orthologue** |
| --- | --- |
| B0250.1 | RPL8 |
| B0336.10 | RPL23 |
| B0393.1 | RPSA |
| B0511.10 | EIF3E |
| C01F1.3 | TGDS |
| C04F12.4 | RPL14 |
| C05C8.7 | MPI |
| C06A1.1 | VCP |
| C06H2.1 | - |
| C07G2.3 | CCT5 |
| C08B11.5 | SF3B4 |
| C09D4.5 | RPL19 |
| C09H10.10 | - |
| C0H6.5 | - |
| C13B9.3 | ARCN1 |
| C14B9.7 | RPL21 |
| C15C8.2 | NPAS4 |
| C17H12.14 | ATP6V1E1 |
| C23G10.3 | RPS3 |
| C24H11.7 | GBF1 |
| C26D10.2 | DDX39B |
| C26E6.4 | POLR2B |
| C26F1.9 | RPL39 |
| C27A2.2a | - |
| C27D11.1 | - |
| C32D5.12 | - |
| C32E8.2 | RPL13 |
| C34B2.10 | SPCS1 |
| C34E10.6 | ATP5B |
| C36B1.4 | PSMA7 |
| C36E8.5 | - |
| C37C3.2 | EIF5 |
| C37C3.3 | CHMP4B |
| C37H5.8 | HSPA9 |
| C39F7.4 | RAB1A |
| C42D8.5 | ACE |
| C44H4.2 | PSMB7 |
| C47B2.3a | - |
| C47B2.4 | - |
| C47E12.5 | UBA6 |
| C49H3.11 | RPS2 |
| C50C3.6 | PRPF8 |
| C50F2.3 | XAB2 |
| C52E4.3 | SNRPD2 |
| C53A5.1 | - |
| C53D5.6 | - |
| C56C10.3 | CHMP4B |
| C56G2.6 | - |
| CD4.6 | PSMA1 |
| D1007.12 | RPL24 |
| D1007.6 | RPS10 |
| D1014.3 | NAPA |
| D1054.15 | PLRG1 |
| D1081.8 | CDC5L |
| E01B7.1 | - |
| E04A4.8 | RPL18A |
| EEED8.5 | DHX8 |
| F01F1.8 | CCT6A |
| F02E8.1 | ATP5F1 |
| F07D10.1 | RPL11 |
| F18A1.3 | - |
| F18C12.2A | - |
| F20B6.1 | - |
| F20B6.2 | ATP6V1B1 |
| F20G4.1 | NBAS |
| F22B5.9 | FARSB |
| F23C8.6 | CHMP1B |
| F25B4.6 | HMGCS1 |
| F25H2.10 | RPLP0 |
| F25H5.4 | EEF2 |
| F26D10.3 | HSPA8 |
| F26E4.8 | - |
| F26H9.6 | RAB5B |
| F28C6.7a | - |
| F28H1.3 | AARS |
| F32H2.6 | - |
| F35H10.4 | ATP6V0A1 |
| F36A2.6 | RPS15 |
| F36A4.7 | POLR2A |
| F37C12.11 | RPS21 |
| F39B2.6 | RPS26 |
| F42C5.1 | - |
| F43D9.3 | SCFD1 |
| F48D6.3 | PTF1A |
| F48F7.1 | - |
| F49C12.12 | - |
| F52B11.3 | - |
| F52C6.3 | UBD |
| F53G12.10 | RPL7 |
| F54C9.5 | RPL5 |
| F54E7.2 | RPS12 |
| F55A3.3 | SUPT16H |
| F56C11.1 | DUOX1 |
| F56F3.5 | RPS3A |
| F56H1.4 | - |
| F57B9.10 | PSMD11 |
| F57B9.2 | CNOT1 |
| F57B9.3 | EIF4A1 |
| F58A4.11 | - |
| F58F12.1 | ATP5D |
| H15N14.1 | ADAD1 |
| H19N07.1 | GSPT1 |
| H28O16.1 | ATP5A1 |
| K01C8.10 | CCT4 |
| K01G5.4 | RAN |
| K02B12.3 | PREB |
| K02D10.5 | SNAP29 |
| K02F2.2 | - |
| K02F2.3 | SF3B3 |
| K04G2.1 | EIF2S2 |
| K05C4.1 | PSMB8 |
| K07D4.3 | PSMD14 |
| K08D12.1 | PSMB6 |
| K10C2.4 | FAH |
| K11H12.2 | RPL15 |
| K12H4.4 | SPCS3 |
| M03F4.2a | - |
| M03F4.6 | - |
| M03F8.3 | CRNKL1 |
| M04B2.1 | - |
| M110.5A | - |
| M28.5 | NHP2L1 |
| R03G5.1 | EEF1A1 |
| R05D11.3 | NUTF2 |
| R12E2.3 | PSMD7 |
| R13A5.12 | PES1 |
| R151.3 | RPL6 |
| T01C3.6 | RPS16 |
| T02H6.11 | UQCRB |
| T04C12.5 | ACTB |
| T05C12.7 | TCP1 |
| T05E11.1 | RPS5 |
| T05F1.3 | RPS19 |
| T05H4.6a | - |
| T07A9.9 | GTPBP4 |
| T08A11.2 | SF3B1 |
| T10B5.5 | CCT7 |
| T13H5.4 | SF3A3 |
| T14F9.1 | ATP6V1H |
| T19B10.2 | - |
| T20F5.2 | PSMB2 |
| T21B10.7 | CCT2 |
| T21E12.4 | DYNC1H1 |
| T23F2.1 | ALG2 |
| T24C4.5 | PRIM1 |
| T27F2.1 | SNW1 |
| T28D9.10 | SNRPD1 |
| VW02B12L.1 | ATP6V0A1 |
| W01D2.2a | - |
| W01F3.3 | - |
| W06F12.1a | - |
| W08E3.1 | SNRPN |
| W08F4.6 | - |
| W09B6.1 | - |
| W09C5.6a | - |
| Y105E8A.9 | AP1G1 |
| Y110A2AL8a | - |
| Y110A7A.8 | PRPF31 |
| Y116A8C.35 | U2AF1 |
| Y116A8C.42 | SNRPD3 |
| Y24D9A.4 | RPL7A |
| Y34D9A.10 | VPS4B |
| Y38A8.2 | PSMB3 |
| Y39G10AR.8 | EIF2S3 |
| Y41D4B.19a | - |
| Y45F10D.12 | RPL18 |
| Y46G5A.4 | SNRNP200 |
| Y46G5A.6 | - |
| Y47D3B.7 | SREBF1 |
| Y47G6A.10 | AFG3L2 |
| Y48G1A.5 | CSE1L |
| Y48G8AL.8 | RPL17 |
| Y49A3A.2 | ATP6V1A |
| Y57G11C.16 | RPS18 |
| Y63D3A.5 | TFG |
| Y71F9AL.12 | - |
| Y71F9B.4 | SNRPG |
| Y71H2B.10 | AP1B1 |
| ZC581.1 | - |
| ZK1236.3 | - |
| ZK328.2 | EFTUD2 |
| ZK430.8 | - |
| ZK622.3a | - |
| ZK652.1 | - |
| ZK652.4 | RPL35 |
| ZK675.1 | PTCH1 |

b) Worm RNAi screen genes modelling tau disease

| **Worm gene** | **Human orthologue** |
| --- | --- |
| B0304.3 | **-** |
| B0310.6 | - |
| B0511.12 | PCNX |
| B0513.6 | - |
| C03B1.2 | - |
| C03G6.5 | - |
| C04B4.2 | - |
| C07G1.5 | HGS |
| C09D4.3 | - |
| C13D9.8 | SLC24A6 |
| C14C11.6 | - |
| C24A11.9 | PDSS1 |
| C24H12.9 | - |
| C30G4.2 | - |
| C32H11.3 | - |
| C37H5.3 | ABHD4 |
| C42C1.15 | ERLIN2 |
| C50E3.12 | - |
| C56E6.1 | - |
| D2030.10 | - |
| E01A2.3 | - |
| F10D2.9 | SCD |
| F11C7.3 | - |
| F21F8.3 | CTSE |
| F26D10.3 | HSPA8 |
| F31B12.2 | - |
| F31D4.3 | FKBP4 |
| F31E8.6 | - |
| F34D6.5 | - |
| F36H1.4 | - |
| F38H4.7 | BTBD2 |
| F38H4.9 | PPP2CA |
| F46G10.3 | SIRT4 |
| F47G4.7 | AMD1 |
| F47G6.1 | DTNA |
| F47G6.3 | - |
| F48E8.5 | - |
| F52B5.2 | - |
| F52C6.6 | - |
| F53B6.4 | - |
| F53F8.1 | - |
| F55C12.7 | - |
| F55F3.2 | - |
| F56H1.5 | AGTPBP1 |
| F59G1.2 | - |
| H14N18.3 | - |
| K09C4.3 | - |
| K09C6.8 | - |
| K10C8.1 | - |
| K12B6.2 | - |
| LLC1.3 | DLD |
| M04G12.3 | - |
| R02D5.3 | - |
| R106.1 | - |
| R13A1.8 | - |
| R74.3 | - |
| T04C9.3 | - |
| T05C12.2 | CHRNA7 |
| T09B4.10 | STUB1 |
| T17E9.1 | TAOK2 |
| T18D3.6 | - |
| T20D3.6 | - |
| T22A3.5 | - |
| T24C4.1 | UQCRC2 |
| T28D9.11 | - |
| W06B11.3 | - |
| Y102A5C.8 | - |
| Y113G7B.4 | - |
| Y18D10A.1 | - |
| Y18D10A.5 | GSK3B |
| Y37A1B.15 | - |
| Y38H8A.3 | - |
| Y52B11B.1 | - |
| Y53C10A.12 | - |
| Y53H1C.3 | - |

c) Worm RNAi screen genes modelling α-synuclein disease

| **Worm gene** | **Human orthologue** |
| --- | --- |
| B0213.12 | - |
| C07A9.8 | BEST2 |
| C07G1.5 | HGS |
| C28C12.7 | - |
| C28H8.11 | TDO2 |
| C28H8.5 | - |
| C34C12.2 | - |
| D2089.1 | SRSF11 |
| E03A3.2 | RECQL5 |
| F10E9.3 | - |
| F21F3.3 | ICMT |
| F26H11.4 | - |
| F28H1.4 | - |
| F29F11.2 | - |
| F32D1.10 | MCM7 |
| F41F3.4 | - |
| F42A10.4 | EEF2K |
| F49B2.6 | - |
| F52C12.2 | TSR3 |
| F52H3.5 | - |
| K07A3.1 | FBP1 |
| K09H9.6 | PPAN |
| M03C11.5 | YME1L1 |
| M151.3 | - |
| R09B3.4 | UBE2F |
| R11A8.4 | SIRT1 |
| R151.6 | DERL2 |
| R151.7 | TRAP1 |
| T05G5.9 | GCC1 |
| T06A4.1 | CPA2 |
| T06G6.8 | - |
| T08H10.1 | - |
| T14F9.1 | ATP6V1H |
| T28F2.2 | - |
| W01B11.2 | - |
| W01B11.6 | - |
| W02A11.2 | VPS25 |
| W02D9.1 | - |
| W03G9.6 | PLA2G7 |
| W05B5.2 | - |
| Y116A8C.35 | U2AF1 |
| Y37H9A.6 | NUDT2 |
| Y48G1A.6 | - |
| Y48G9A.10 | - |
| Y54E2A.2 | SMG9 |
| Y6B3B.10 | CERS1 |
| Y71G12B.20 | - |
| ZK993.2 | - |
